# Vaccination coverage rates and predictors of HPV vaccination among eligible and non-eligible female adolescents at the Brazilian HPV vaccination public program

**DOI:** 10.1186/s12889-020-08561-4

**Published:** 2020-04-06

**Authors:** Alexandre Faisal-Cury, Renata Bertazzi Levy, Maria Fernanda Tourinho, Alexandre Grangeiro, José Eluf-Neto

**Affiliations:** Department of Preventive Medicine Department, University of São Paulo School of Medicine, Avenida Dr Arnaldo 455, São Paulo, 01246-903 Brazil

**Keywords:** HPV vaccine, Adolescents, Prevention of cervical cancer, Inequality, Risk factors

## Abstract

**Background:**

Since March 2014, the quadrivalent HPV vaccine has been incorporated into the Brazilian Unified Health Care System and began to be offered, without direct costs, for girls from 9 to 13 years of age. Older female adolescents would have the option to be vaccinated at private health care system being responsible for the payment of HPV vaccine. The present study aimed to evaluate the coverage rates and predictors of HPV vaccination in Brazil among two groups of female adolescents: eligible and non-eligible for the HPV vaccination public program.

**Methods:**

We used data from the 2015 Brazilian National Adolescent School-Based Health Survey, which involved a probabilistic sample of 5404 female adolescents students at public and private schools. Using a questionnaire, we gathered information on sociodemographic characteristics, sexual behavior, and respondent perception of parental supervision and have been vaccinated for HPV. Age-specific vaccination rates were analyzed in girls aged 9 to 13 at the time of public vaccination (eligible for public policy), as well among those 14 to 17 years old not eligible by the Ministry of Health for vaccination. We used Poisson regression models to investigate associated factors.

**Results:**

HPV vaccine coverage was 83.5 and 21.8% among eligible and non-eligible populations, respectively. In both populations, the chance of being vaccinated decreased with older age. In the eligible population there is a greater chance of being vaccinated among ethnic group “pardas” but not with other indicators of socioeconomic status. In the non-eligible population, there was a clear association between higher vaccine coverage and greater maternal education and living with the mother.

**Conclusion:**

Our findings highlight the importance of public policies to minimize inequities in access to cancer prevention measures in vulnerable adolescents. A public policy of HPV vaccination for older female adolescents would increase coverage with possible reduction of HPV-related diseases in this group of women.

## Background

Excluding non-melanoma skin cancer, cervical cancer is the one of the most common tumors in females in Brazil, second only to breast cancer, and is the fourth leading cause of cancer-related death among women in the country [1]. The National Cancer Institute of Brazil estimates that, in 2016, there were 16,340 new cases of cervical cancer and more than 5000 deaths from the disease in Brazil [2]. Almost all cases of cervical cancer, as well as many cases of cancer of the vulva, vagina, penis, and oropharynx, are associated with persistent infection by oncogenic types of human papillomavirus (HPV), especially HPV 16 and 18 [3].

Vaccination against HPV is an effective primary prevention strategy to reduce HPV infection [4, 5]. Since the approval of the HPV vaccine by the United States Food and Drug Administration in 2006, HPV vaccination programs have been progressively introduced in several countries, typically targeting girls between 9 and 14 years of age. Since 2006, the HPV vaccine has been registered in Brazil and made available at private clinics and laboratories in the country, HPV vaccination being recommended for females between 9 and 26 years of age according to the vaccine producer. In March 2014, the quadrivalent HPV vaccine was incorporated into the Brazilian Unified Health Care System and began to be offered, without direct costs, at primary care clinics, public schools, and private schools for girls from 9 to 13 years of age. The Brazilian government set a goal of vaccinating 80% of the 5.2 million girls in this age group in the country. Female adolescents between 9 and 13 years of age were defined as the target population for HPV vaccination and would receive a vaccination at public health system without any costs. Older female adolescents would have the option to be vaccinated at private health care system being responsible for the payment of HPV vaccine. To reach this goal, mass campaigns aimed at the general population, health professionals, and adolescents were carried out by the Brazilian government. Studies have confirmed that vaccination against HPV is cost-effective in Brazil, especially if high vaccination coverage rates can be achieved [6].

Vaccination coverage varies widely among countries, being higher in high-income countries. A systematic review of studies conducted between 2006 and 2014, including data from 64 countries, showed that the proportion of females between 10 and 20 years of age who received a complete course (three doses) of the HPV vaccine was higher in high-income countries than in middle- and low-income countries (33.6% vs. 2.7%) [7]. However, even in developed countries, such as the United States, HPV vaccination coverage among adolescent girls is low compared with that for other types of vaccination, such as those against tetanus and meningococcal infection. By 2015, 62.8% of girls 13–17 years of age in the United States had received at least one dose of the vaccine, whereas 34.8% had received all three doses [8].

Various factors have been associated with non-vaccination, including national priorities and fundings, access to healthcare, socioeconomic factors, as well as the attitudes of and knowledge on the part of health care professionals and relatives or caregivers [9–13]. In addition a number of factors influence acceptance of the HPV vaccine by relatives and caregivers: cultural and religious perceptions about sexual activity [14]; recognition that the vaccine is safe and effective [15]; fear of regretting the decision not to vaccinate; adherence to other routinely recommended vaccinations; social norms; and a positive influence from the media [16]. To our knowledge, there are no studies addressing the association between vaccination and the perception that young people have of the care provided by their parents. Attention provided by relatives could possible improve the health care of adolescents.

In the United States, studies on the association between HPV vaccination and family income and between HPV vaccination and the type of health care have produced controversial results. One such study showed that adolescents from lower-income families without health insurance are more likely to initiate the HPV vaccination process [17]. However, other studies of HPV vaccination have found that the health insurance status is not associated with vaccination or with the intention to vaccinate [18, 19]. Nevertheless, Liddon et al. [20] showed that vaccination against HPV in females 15–19 years of age is associated with having health insurance, although not with ethnicity or socioeconomic status. There are also discrepancies among studies regarding the association between HPV vaccination and sexual behavior. A nationwide survey in the United States found that, among adolescent girls between 14 and 18 years of age, the proportion of girls vaccinated against HPV was greater among those who had become sexually active than among those who had not (28.6% vs. 17.8%) [21]. In another such survey, the National Survey of Family Growth, which involved 1243 females 15–24 years of age, vaccination was not found to be associated with sexual activity or with the number of sexual partners [22]. As for the public vaccination strategy, school-based HPV immunization programs have been shown to produce higher rates of coverage than do the corresponding clinic-based programs [23, 24]. In Brazil, the HPV vaccination program launched in 2014 was carried out mainly in schools focusing only female adolescents.

Studying HPV vaccination coverage reached in various contexts is critical to assessing the benefits of different vaccination strategies and identifying possible disparities in access. The present study aimed to analyze HPV vaccination coverage in populations of adolescent girls to whom the vaccine was available via the Unified Health Care System, without direct costs (eligible), and those who were older and not eligible for the Brazilian HPV vaccination program. In both scenarios, we also evaluated the predictors of vaccination.

## Methods

The present study used data from the third edition of the Brazilian National Adolescent School-Based Health Survey—PeNSE 2015—conducted by the Brazilian Institute of Geography and Statistics, in partnership with the National Ministry of Health and Education [25] between April and September 2015. The study involved a representative sample of female students, between 13 and 17 years of age, selected from among 6th–12th grade students at public and private schools in representative urban and rural areas of Brazil.

In this survey, a secondary data analysis, we employed a complex clustered sampling procedure, first selecting schools (primary sampling units) in each of the major regions and then selecting classes (secondary sampling units) in those schools. All of the students in those classes were invited to participate in the survey. The sampling weights for schools and students were adjusted on the basis of the 2015 school census, which contained up-to-date registry information. The response rate was 85.3%. The final sample included 16,608 students in 653 classes at 371 schools. Only data from girls (*N* = 8269) were used in the present study.

From among all of the girls (*N* = 8269), we selected a representative sample of those who were 13–17 years of age (*n* = 5404). Detailed information about the sampling strategy can be found in the report published by the Brazilian Institute of Geography and Statistics [25].

The data were collected with a smartphone provide by the research team that presented an electronic, self-report questionnaire.^1^ The questions addressed a variety of subjects: sociodemographic characteristics; family situation; eating habits and nutrition; physical activity; tobacco, alcohol and illicit drug use; sexual, reproductive, and mental health; history of violence/abuse, risky behavior, and accidents; personal hygiene; body image; and anthropometric variables.

In the present study, the guidelines established by the Brazilian National Ministry of Health (NMH) for HPV vaccination were used in order to stratify the adolescents by the type of access to vaccination (eligible and non-eligible). Therefore, girls 9–13 years of age at the time of vaccination, who were the target population of the NMH, were considered eligible population, whereas females between 13 and 17 years of age at the time of vaccination were considered the non-eligible for the public vaccination. To calculate the age of the girls at the time of the vaccination, we took into consideration the months (March and September 2014 and 2015) that the NMH campaigns for to promote public vaccination and the day of the interview, together with the month and year of birth.

### Outcome measure

The outcome measure was having received the vaccine against HPV, evaluated by means of the question “Have you been vaccinated against HPV?”

### Factors associated with vaccination

On the basis of the PeNSE 2015 questionnaire data, the following sociodemographic factors were assessed: age of the respondent (13 to 17 years of age); ethnic group (White, Black, Asiatic, Parda or Indigenist); maternal level of education (< 9 years of schooling, 9 years of schooling, 9–11 years of schooling, 12 years of schooling, some college, or college graduate); type of school (public or private); and consumer goods and services score for the family (evaluated in tertiles). The consumer goods and services score was based on whether the family had an automobile, a home telephone, a cell phone, a computer, or internet access, as well as whether there was a bathroom in the home and whether the family had a maid in five or more days per week. Each of those items were given a weight that was the inverse of the frequency of their possession or presence in the sample as a whole. The score for each adolescent was obtained by summing the weights of the respective items. We divided the distribution of the score into thirds, considering the effect of complex sampling [26]. The sexual behavior of the respondents was assessed by collecting the following data: history of sexual intercourse (yes or no); number of sexual partners (0, 1, or ≥ 2); age at first intercourse (not applicable, 9–11 years, 12–14 years, or 15–17 years); condom use at first intercourse (not applicable, yes, or no); and condom use in the most recent sexual encounter (not applicable, yes, or no). Due to the high correlation among the variables related to sexual behavior, we chose to use only the number of sexual partners as a variable in the final model.

Multiple imputation by chained equations was used in order to assign numerical values to the maternal level of education, for which 20% of the data were missing. The imputed data exhibited satisfactory statistical reproducibility according to error analysis by Monte Carlo simulation [27].

We evaluated the following aspects of parental care among the respondents: living with parents (both, the mother only, the father only, or neither) and perception about parental involvement (parents almost always/always or never/rarely/sometimes being attentive to the problems and concerns of the respondent).

### Statistical analysis

The HPV vaccination coverage, expressed as a proportion and its 95% CI, was estimated on the basis of the total number of respondents, by the variables studied and according to the type of access to vaccination (eligible population and non-eligible population). To estimate the association between HPV vaccination coverage and each of the selected variables, the prevalence ratios (PRs) and respective 95% CIs were calculated. Unadjusted and adjusted Poisson regression models, stratified by the type of access to HPV vaccination, were used in order to assess the associations between HPV vaccination coverage and each of the selected variables adjusted for the other independent variables. Poisson regression was used in order to prevent odds ratio overestimating risk considering the high rate of the outcome. In the bivariate Poisson regression models of the associations between the dependent variable (HPV vaccination coverage) and the independent variables, all covariates were included. In the multivariate analysis, values of *P* < .05 were considered statistically significant. The models used in the analysis of the linear trend included the following as continuous variables: the maternal level of education; the consumer goods and services score of the family; and the number of sexual partners of the respondent. In all analyses, we took into consideration the sampling design, with the aid of the Stata statistical software package, version 15.1 (StataCorp, College Station, Texas).

### Ethical aspects

The PeNSE was approved by the NMH National Committee for Ethics in Research (Protocol no. 1006467). Student participation was voluntary. All participants gave informed consent through a self-administered questionnaire. The questionnaire could be answered in whole or in part. All information was confidential and anonymous.

## Results

### Demographic and behavioral characteristics

The study included 5404 adolescent females between 13 and 17 years of age. In the sample as a whole, the proportions of respondents eligible and non-eligible for the public vaccination were 44.0 and 56.0%, respectively. In the eligible and non-eligible populations, public school students predominated (accounting for 87.2 and 86.2%, respectively), as did non-White students (66.1 and 63.2%, respectively). In the eligible population, 47.7 and 33.9% of the respondents described their ethnic group as “parda” and White, respectively; 13.6% had had sexual intercourse at least once; 54.9% lived with both parents; and 65.8% reported having parents that were almost always/always aware of where their children were. In the non-eligible population, 46.2 and 36.8% of the respondents described their ethnic group as “parda” and White, respectively; 44.9% had had sexual intercourse at least once; 22.0% had had ≥2 sexual partners; and 27.0% had not used condoms in their most recent sexual encounter (Table 1).

**Table 1:** Potential predictive factors, in relation to the characteristics of and HPV vaccination rates for the eligible and non-eligible populations of female 9th-grade students in Brazil - PeNSE 2015 data

	Population characteristics			HPV Vaccination rates		
	Total	Eligible population (%)	Non-Eligible population (%)	Total	Eligible population (%)	Non-Eligible population (%)
Total	5.404	44,0	56,0	48,9	83,5	21,8
Age (years)						
13	19,4	44,1	–	89,4	89,4	–
14	20,2	45,8	–	79,8	79,8	–
15	21,5	10,2	30,5	39,0	74,0	29,8
16	21,1	–	37,6	18,1	–	18,1
17	17,8	–	31,9	18,4	–	18,4
Ethnic group						
White	35,5	33,9	36,8	46,6	79,9	22,4
Black	10,4	10,9	10,0	51,8	84,0	24,2
Asiatic	4,7	4,3	5,1	43,7	81,8	18,3
Parda	46,9	47,7	46,2	50,2	86,2	21,0
Indigenist	2,5	3,2	2,0	56,3	79,5	24,6
Maternal level of education						
< 9 years of schooling	35,6	36,9	34,7	48,3	82,3	19,9
9–11 years of schooling	16,8	18,0	15,8	48,6	82,4	18,4
Some college	31,5	28,6	33,8	45,7	84,0	20,3
College graduate	16,0	16,5	15,7	56,6	86,0	32,4
Consumer goods and services score (tertiles)						
Lower	38,0	40,8	35,8	48,7	82,4	18,6
Medium	29,8	28,5	30,8	47,5	83,2	21,4
Higher	32,2	30,7	33,5	50,5	84,9	25,6
Type of school						
Private	13,4	12,8	13,8	52,3	86,2	27,9
Public	86,6	87,2	86,2	48,4	83,1	20,8
Has had sexual intercourse						
No	68,9	86,5	55,1	56,3	83,9	22,2
Yes	31,1	13,6	44,9	32,7	80,3	21,3
Number of sexual partners						
0	69,1	86,5	55,3	56,3	83,9	22,1
1	16,3	8,2	22,7	35,1	84,9	21,0
2 or more	14,6	5,3	22,0	29,7	73,4	21,3
Age at first intercourse (years)						
Not applicable	69,1	86,6	55,4	56,3	83,9	22,1
9 to 11	0,7	0,8	0,6	56,0	88,0	23,4
12 to 14	14,8	12,2	16,9	45,5	80,1	25,7
15 to 19	15,4	0,5	27,1	19,0	73,6	18,3
Condom use in most recent encounter						
Not applicable	69,3	86,9	55,5	56,3	83,9	22,1
Yes	11,9	4,9	17,5	31,0	86,2	18,8
No	18,8	8,2	27,0	33,4	76,9	22,8
Condom use at first intercourse						
Not applicable	69,1	86,5	55,4	56,3	83,9	22,1
Yes	8,8	4,9	11,9	32,1	81,7	15,8
No	22,1	8,6	32,8	32,8	79,7	23,0
Living/not living with parents						
Living with both parents	54,9	54,9	55,0	49,4	83,5	22,7
Living with the mother only	33,1	33,5	32,9	49,6	83,3	22,5
Living with the father only	4,2	4,4	4,0	46,8	87,5	11,5
Living with neither parent	7,8	7,3	8,1	44,1	82,1	17,4
Parents attentive to the problems and concerns of the respondent						
Almost always/always	43,7	42,5	44,6	49,4	83,6	23,8
Never/rarely/sometimes	56,3	57,5	55,4	48,5	83,2	20,1

### Vaccination rate

In the sample as a whole, the HPV vaccination coverage was 48.9%. In the eligible and non-eligible populations, the coverage was 83.5 and 21.8%, respectively. In the eligible population, vaccination rates were 89.4% among those who were 13 years old at time of vaccination and 88.0% among those who had first sexual intercourse between 9 and 11 years of age. The lowest vaccination rates in the eligible population were among the respondents who had at least two sexual partners (73.4%), who had first sexual intercourse after 15 years of age (73.6%) and among those who were 15 years old at the time of the vaccination campaign (74.0%). In contrast, vaccination rates among non-eligible population ranged from 32.4%, for the respondents whose mother had college degree to 11.5%, for those who lived with their father only (Table 1).

### Predictors

In the eligible population, the following self-reported characteristics were associated with HPV vaccination coverage (Table 2): being 14 years of age at the time of vaccination in comparison with being 13 years of age (PR:0.89, 95% CI 0.84–0.94); being 15 years of age in comparison with being 13 years of age (PR:0.83, 95% CI 0.76–0.92); and being “parda” in comparison with being white (PR:1.09, 95% CI 1.02–1.16).

**Table 2:** HPV vaccination coverage in the eligible population, in relation to the potential predictor factors. Prevalence Ratios (PR), crude and adjusted, with 95% confidence interval (95% CI)

	Eligible Population					
	PR crude	95% CI		PR _adjusted_	95% CI	
Age (years)						
≤ 13	1,00			1,00*		
14	0,892	0,846	0,942	0,891	0,844	0,940
15	0,828	0,752	0,911	0,835	0,757	0,920
Ethnic group						
White	1000			1000		
Black	1051	0,979	1129	1052	0,979	1131
Asiatic	1023	0,887	1180	1015	0,877	1176
Parda	1079	1013	1149	1087	1017	1162
Indigenist	0,995	0,845	1171	0,989	0,841	1162
Maternal level of education						
< 9 years of schooling	1000			1000		
9–11 years of schooling	1001	0,915	1094	1004	0,917	1099
Some college	1020	0,930	1120	1017	0,921	1124
College graduate	1046	0,968	1129	1030	0,939	1131
Consumer goods and services score (tertiles)						
Lower	1000			1000		
Medium	1009	0,950	1071	1019	0,958	1083
Higher	1030	0,977	1087	1041	0,978	1110
Type of school						
Private	1000			1000		
Public	0,964	0,896	1037	1002	0,920	1090
Has had sexual intercourse						
No	1000			–	–	–
Yes	0,957	0,884	1036	–	–	–
Number of sexual partners						
0	1000			1000		
1	1012	0,932	1098	1027	0,941	1120
2 or more	0,875	0,755	1015	0,899	0,768	1053
Age at first intercourse (years)						
Not applicable	1000			–	–	–
9 to 11	1049	0,926	1187	–	–	–
12 to 14	0,954	0,878	1038	–	–	–
15 to 19	0,877	0,602	1278	–	–	–
Condom use in most recent encounter						
Not applicable	1000			–	–	–
Yes	1027	0,927	1137	–	–	–
No	0,916	0,818	1026	–	–	–
Condom use at first intercourse						
Not applicable	1000			–	–	–
Yes	0,973	0,863	1096	–	–	–
No	0,950	0,853	1057	–	–	–
Living/not living with parents						
Living with both parents	1000			1000		
Living with the mother only	0,998	0,938	1062	1010	0,947	1076
Living with the father only	1048	0,953	1153	1049	0,951	1156
Living with neither parent	0,984	0,889	1088	0,991	0,896	1096
Parents attentive to the problems and concerns of the respondent						
Almost always/always	1000			1000		
Never/rarely/sometimes	0,995	0,951	1041	0,998	0,953	1045

*p trend < 0.001

In the non-eligible population, the following self-reported characteristics were associated with HPV vaccination coverage (Table 3): being 16 or 17 years of age at the time of vaccination in comparison with being 15 years of age (PR:0.62, 95% CI 0.49–0.78); having a mother who had college degree in comparison with a mother who had an incomplete elementary school degree (PR:1.42, 95% CI 1.04–1.94); and living only with the father in comparison with living with both parents (PR:0.51, 95% CI 0.31–0.85).

**Table 3:** HPV vaccination coverage in the non-eligible population, in relation to the potential predictor factors. Prevalence Ratios (PR), crude and adjusted, with 95% confidence interval (95% CI)

	Non-eligible population					
	PR crude	95% CI		PR _adjusted_	95% CI	
Age (years)						
≤ 15	1000			1,00*		
16	0,608	0,483	0,765	0,615	0,487	0,777
17	0,617	0,486	0,784	0,617	0,489	0,779
Ethnic group						
White	1000			1000		
Black	1078	0,795	1464	1220	0,914	1628
Asiatic	0,815	0,534	1244	0,888	0,573	1376
Parda	0,937	0,787	1114	1031	0,868	1224
Indigenist	1096	0,620	1938	1202	0,659	2191
Maternal level of education						
< 9 years of schooling	1000			1000		
9–11 years of schooling	0,924	0,662	1289	0,923	0,671	1271
Some college	1020	0,783	1329	0,953	0,733	1239
College graduate	1627	1233	2147	1419	1040	1937
Consumer goods and services score (tertiles)						
Lower	1000			1000		
Medium	1154	0,846	1573	1138	0,832	1557
Higher	1376	1044	1813	1190	0,883	1603
Type of school						
Private	1000			1000		
Public	0,744	0,592	0,935	0,918	0,709	1189
Has had sexual intercourse						
No	1000			–	–	–
Yes	0,959	0,804	1144	–	–	–
Number of sexual partners						
0	1000			1000		
1	0,947	0,757	1185	1055	0,855	1301
2 or more	0,963	0,759	1222	1089	0,845	1403
Age at first intercourse (years)						
Not applicable	1000			–	–	–
9 to 11	1060	0,389	2887	–	–	–
12 to 14	1162	0,919	1471	–	–	–
15 to 19	0,825	0,661	1032	–	–	–
Condom use in most recent encounter						
Not applicable	1000			–	–	–
Yes	0,850	0,662	1091	–	–	–
No	1033	0,830	1286	–	–	–
Condom use at first intercourse						
Not applicable	1000			–	–	–
Yes	0,715	0,514	0,995	–	–	–
No	1042	0,870	1247	–	–	–
Living/not living with parents						
Living with both parents	1000			1000		
Living with the mother only	0,990	0,762	1286	1044	0,804	1357
Living with the father only	0,505	0,306	0,832	0,509	0,307	0,846
Living with neither parent	0,766	0,540	1088	0,851	0,592	1223
Parents attentive to the problems and concerns of the respondent						
Almost always/always	1000			1000		
Never/rarely/sometimes	0,847	0,707	1014	0,860	0,713	1038

*p trend < 0.001

## Discussion

The HPV vaccination coverage is high in Brazil, being 83.5% among young girls who are the target of the public vaccination strategy and are offered the vaccine, without direct payment, via the immunization program of the NMH, mainly in schools. In this group, we found that a social characteristic, the “pardas” ethnic group, was associated with vaccine coverage against HPV. In Brazil, “pardas” encompass people who use racial miscegenation to self-refer their skin color/ethnic group (black-white, black- indigenous, white-black- indigenous, etc.). Among the interviewed girls, who were not eligible for the HPV vaccination public program, vaccine coverage reduced to 21.8% and was associated with parental care of the mother and higher schooling. In this group, girls were more likely to be vaccinated if they lived with their mothers and if their mothers had a higher level of schooling. These findings emphasize that, in the absence of universal public policies, adolescents living only with the father and inserted in a context of greater social vulnerability will have reduced access to vaccination and will be more subject to health problems, such as cervical cancer.

In the eligible and non-eligible populations, our results showed a clear association between higher vaccination rates and younger age. One possible explanation for that is that the benefit of vaccination has become more evident in recent years in the light of scientific evidence and media campaigns. Therefore, younger generations are more likely to be vaccinated.

Another important aspect common to both of the populations studied is the lack of association between HPV vaccination and indicators of perception of parental care. The chance of being vaccinated against HPV was not significantly different according to the perception of the respondents about parental concern and attention. Despite the large number of studies emphasizing the importance of parental attitudes and beliefs regarding HPV immunization, we identified no studies specifically addressing the perceptions that adolescents have of parental care.

Nevertheless, our results show that mothers have a significant influence on the rates of vaccination among girls not covered by the public program. In this group, girls living only with their father were less likely to be vaccinated than were those living with both parents or those living only with the mother. These data suggest that mothers are more concerned than are fathers or have more influence on HPV vaccination of their daughters. That type of concern is enhanced by a higher maternal level of education. Having a more well-educated mother increases the chance that a daughter will be vaccinated. Mother’s higher education could also be associated with knowledge (and then concern) about HPV and ability to pay for the vaccine. To our knowledge, there are no other studies comparing mothers and fathers in terms of the role they play in HPV vaccination.

Our data on vaccination coverage are quite encouraging, given that national and international studies have suggested that vaccination coverage above 70% is more cost-effective [6, 28, 29]. Studies involving smaller populations and conducted in certain cities in Brazil have reported high HPV vaccination rates [30] However, the present study involved a sample of 13–17 years old students residing in cities of various profiles, in all regions of the country. That gives our study high power to analyze the success of national HPV vaccination strategies and policies. Thus, it calls attention to the high HPV vaccination coverage, especially considering the short period of implementation of the program (2 years), the fact that Brazil is a country of continental dimensions, and the barriers to equal access to health care services in the country [31].

However, the HPV vaccination coverage for young females who were not included in HPV vaccination public program is much less encouraging. Because they were not included in the national vaccination campaigns, only 21.8% of those girls were vaccinated against HPV, seeking vaccination spontaneously or being vaccinated because they were influenced by their parents and schools. It should be borne in mind that the Brazilian Unified Health Care System does not provide vaccination for individuals who are outside the recommended age ranges. Such individuals must seek vaccination at private clinics. Since their inception, campaigns promoting vaccination against HPV have been carried out in various media and at health care clinics, focusing on the general population and, specifically, girls 9–13 years of age, although also touting the benefits of immunization against HPV in older females, not only for the prevention of various types of cancer (cervical and penile) but also for the prevention of genital warts.

From a collective point of view, the HPV vaccination strategy, focusing only on girls between 9 and 13 years of age, accentuates a pattern of inequality in older females who do not receive adequate protection against HPV. It is of note that the new NMH guidelines, issued in 2017, are aimed at partially correcting this distortion, expanding the program to include 14-year-old girls who have not yet been vaccinated or who did not complete the three-dose cycle, as well as boys 12–13 years of age.^2^ The strategy to vaccinate boys, unprecedented in South America, has already been adopted in the United States, Australia, Austria, Israel, Puerto Rico, and Panama. The strategy has the support of several scientific societies [32] as well as that of the U.S. Centers for Disease Control and Prevention Advisory Committee on Immunization Practices [33].

In the non-eligible population, the only socioeconomic indicator found to be associated with higher HPV vaccination coverage was the maternal level of education. That suggests that a higher level of education enhances the care provided by the mother. One previous study showed that the daughters of women with a lower level of education are less likely to be vaccinated [34], which could be explained by the lower levels of knowledge and confidence such mothers have about the safety and benefits of immunization against HPV [35]. According to one qualitative systematic review, interventions to overcome barriers to HPV vaccination should also focus on the issue of trust, which requires providing direct, accessible, and, in some cases, culturally appropriate information on vaccination programs [14]. Additionally, maternal education is an indicator of socioeconomic status that has been recognized as a very important barrier to vaccination access even in developed countries.

In the United States, the cost of vaccination is considered as an important barrier to the initiation and completion of the vaccination cycle [9, 36, 37] Historically, women of lower socioeconomic status are less likely to initiate and complete the HPV vaccination process [37, 38]. A systematic review of 22 studies showed that cost is the principal factor impeding access to the vaccine [39]. However, according to a meta-analysis, lack of health insurance is even more important than is income as a factor associated with non-vaccination [38] a finding that has profound implications for vaccination strategies.

The results of the present study show that among young females with access to the HPV vaccine via a public health care system there was no association between vaccine rate and number of sexual partners. In the literature, there are divergent data on the relationship between vaccination and sexual behavior, reflecting differences across studies in terms of the characteristics of the samples, such as age, social status, and cultural norms, as well as different outcome measures for sexual behaviors and methodological differences. Some studies have suggested that young females who have been vaccinated against HPV have fewer sexual partners [18, 40] whereas other studies have found that HPV vaccination is not associated with the number of sexual partners or with the age at first intercourse [22, 41–43].

Among the strengths of the present study are the fact that it involved a broad population-based sample, obtained through a probabilistic sampling process, and the unprecedented social, behavioral, and parental/school monitoring/care aspects. Another strength is the analysis of data from two different female populations, eligible and non-eligible.

One limitation to be considered in the present study is that the HPV vaccination coverage was evaluated through a direct questionnaire, although it is common practice in studies of this type. That might have increased the risk of non-differential misclassification in the analysis of vaccination coverage rates, because young people could forget or be confused about which vaccine they have previously taken. However, 95.6% of the young women in our sample showed a high degree of knowledge about the HPV vaccine, as assessed with a specific question on the questionnaire (results not shown). In addition, the response rate was, in general, quite high (95%). However, for the maternal education variable, the non-response rate was 24% and the data were imputed. That increased the reliability and power of the analysis [44]. Although there might have been an information bias for issues related to sexuality and sexual activity, the anonymity of the responses minimizes that possibility [45] In addition, self-reporting reduces respondent exposure to the interviewer on sensitive issues and can thus provide information that is more valid and reliable than that obtained in a face-to-face interview [46]. Another aspect is that we evaluated only the history of having received the HPV vaccine, without defining the number of doses, which limited our ability to characterize the immunological status of this population. However, there is evidence that once the vaccination cycle has been initiated there is a high chance that it will be completed (i.e., all three doses will be given) [30]. In addition, recent studies have shown that efficacy/effectiveness is similar among 1, 2, and 3 doses [47]. Two additional facts support the use of one single dose: younger adolescents achieve similar level of protection regardless the number of doses received and a single dose provides considerable protection compared with no vaccination at al [48]. A further limitation is the assumption that providers adherence to the age criteria is strict. We cannot exclude the possibility that female adolescents over 13 years old manage to obtain the vaccination in the public sector. On the other hand, it is quite unlikely that eligible girls opt to receive the HPV vaccination in the private sector where they have to pay. Therefore, if this were the case, the difference of coverage between non-eligible and eligible groups would be even higher. Regarding external validity, the PeNSE 2015 study included a representative sample of 13–17 girls from public and private schools in Brazil, which limits extrapolation of the results to young people in other age groups and to nonstudents. However, the rate of enrollment in elementary and secondary school is quite high in Brazil (97.7 and 84.3%, respectively) [49].

## Conclusions

The present study presents an overview of HPV vaccination coverage in Brazil, providing elements to better understand the scope and possible limitations of universal public and non-universal access vaccination strategies. Monitoring the outcomes and coverage of the HPV immunization program, together with management of technology transfer and the development of electronic vaccination records, are key elements in the long-term success of the public policy [50]. Public health measures and the evaluation of immunization strategies to promote the health of young people depend on the recognition of gaps and the management of factors associated with possible inequities in vaccination coverage. However, the current vaccination rate in Brazil is quite high for young girls (9–13 years of age) who are covered by the public HPV immunization program, which is not the case for older females, who not have access to the public vaccination. A high proportion of the latter group remains particularly susceptible to HPV-related diseases, especially cervical cancer. A public policy of HPV vaccination for older female adolescents would increase coverage with possible reduction of HPV-related diseases in this group of women.

## Data Availability

The public access to database is open. Database is available on the link below. https://www.ibge.gov.br/estatisticas/sociais/populacao/9134-pesquisa-nacional-de-saude-do-escolar.html?=&t=downloads

## Abbreviations

HPV: Human papillomavirus
NMH: Brazilian National Ministry of Health
PeNSE 2015: Brazilian National Adolescent School-Based Health Survey—PeNSE 2015
PRs: Prevalence ratios

## Footnotes

1 (https://biblioteca.ibge.gov.br/visualizacao/instrumentos_de_coleta/doc4595.pdf)

2 (http://portalsaude.saude.gov.br/index.php/o-inisterio/principal/secretarias/svs/noticias-svs/25991-meninos-tambem-serao-vacinados-contra-hpv).
